# How COVID-19 Affected the Journal Impact Factor of High Impact Medical Journals: Bibliometric Analysis

**DOI:** 10.2196/43089

**Published:** 2022-12-21

**Authors:** Orestis Delardas, Panagiotis Giannos

**Affiliations:** 1 Promotion of Emerging and Evaluative Research Society London United Kingdom; 2 Department of Life Sciences Faculty of Natural Sciences Imperial College London London United Kingdom

**Keywords:** COVID-19, journal impact factor, scientometrics, bibliometrics, infometrics, journal, assessment, research, resources, medical journal, literature, database, community, behavior

## Abstract

**Background:**

Journal impact factor (IF) is the leading method of scholarly assessment in today’s research world, influencing where scholars submit their research and where funders distribute their resources. COVID-19, one of the most serious health crises, resulted in an unprecedented surge of publications across all areas of knowledge. An important question is whether COVID-19 affected the *gold standard of scholarly assessment*.

**Objective:**

In this paper, we aimed to comprehensively compare the productivity trends of COVID-19 and non–COVID-19 literature as well as track their evolution and scholarly impact across 3 consecutive calendar years.

**Methods:**

We took as an example 6 high-impact medical journals (Annals of Internal Medicine [Annals], The British Medical Journal [The BMJ], Journal of the American Medical Association [JAMA], The Lancet, Nature Medicine [NatMed], and The New England Journal of Medicine [NEJM]) and searched the literature using the Web of Science database for manuscripts published between January 1, 2019, and December 31, 2021. To assess the effect of COVID-19 and non–COVID-19 literature in their scholarly impact, we calculated their annual IFs and percentage changes. Thereafter, we estimated the citation probability of COVID-19 and non–COVID-19 publications along with their rates of publication and citation by journal.

**Results:**

A significant increase in IF change for manuscripts including COVID-19 published from 2019 to 2020 (*P*=.002; Annals: 283%; The BMJ: 199%; JAMA: 208%; The Lancet: 392%; NatMed: 111%; and NEJM: 196%) and to 2021 (*P*=.007; Annals: 41%; The BMJ: 90%; JAMA: 6%; The Lancet: 22%; NatMed: 53%; and NEJM: 72%) was seen, against non–COVID-19 ones. The likelihood of highly cited publications was significantly increased in COVID-19 manuscripts between 2019 and 2021 (Annals: *z*=3.4, *P*<.001; The BMJ: *z*=4.0, *P*<.001; JAMA: *z*=3.8, *P*<.001; The Lancet: *z*=3.5, *P*<.001; NatMed: *z*=5.2, *P*<.001; and NEJM: *z*=4.7, *P*<.001). The publication and citation rates of COVID-19 publications followed a positive trajectory, as opposed to non–COVID-19. The citation rate for COVID-19 publications peaked by the second quarter of 2020 while that of the publication rate approximately a year later.

**Conclusions:**

The rapid surge of COVID-19 publications emphasized the capacity of scientific communities to respond against a global health emergency, yet inflated IFs create ambiguity as benchmark tools for assessing scholarly impact. The immediate implication is a loss in value of and trust in journal IFs as metrics of research and scientific rigor perceived by academia and society. Loss of confidence toward procedures employed by highly reputable publishers may incentivize authors to exploit the publication process by monopolizing their research on COVID-19 and encourage them to publish in journals of *predatory* behavior.

## Introduction

The COVID-19 pandemic has presented unique challenges to modern societies. The spread of SARS-CoV-2 worldwide during the early 2020 posed unprecedented disruption in human lives and significant stresses to public health structures and socioeconomic systems [1]. *Feverish* academic activity on COVID-19 research has been recorded across all areas of knowledge with almost immediate effects since the disease was discovered in December 2019. Surges in COVID-19 infections in China and Italy initiated the first wave of COVID-19 publications within the first 3 months of the pandemic [2]. The die had been cast.

From an early stage, it was evident that the rate of publications was the most of any disease published thus far; however, very few constituted high-level original research including meta-analyses, systematic reviews, or control trials while preprints, opinion pieces, editorials or commentaries filled the void [2,3]. Adding to the exponential growth in COVID-19 publications was also the expedited editorial peer-review processes put in place for manuscripts that generated record speeds in processing times and article acceptance [4]. Indeed, Palayew et al [5] revealed that median time from submission to acceptance of COVID-19 research was reduced to 6 days from 84 days when compared to non–COVID-19 content during the first months of 2020. Horbach [6] further showed that the majority of this decrease was attributed to an acceleration of the review process.

These steps of disproportionately shorter-than-usual processes taken by academic journals reflect the particular urgency for information-sharing and altmetric dissemination across scientific fields [7]. However, fast-track review methods and processes developed during the COVID-19 pandemic are “here to stay” [8]. Although reasonable to expect such policies during times of extraordinary mobilization for novel treatments and best practices to combat a disease of this scale, not all outcomes are rosy. Much as the COVID-19 infodemic might have been paved with good intentions, efforts to loosen the demanding process of peer reviewing can seriously undermine research quality and the potential merit of journals in their attempt.

Critiques of such approaches have been described in the literature. Palayew et al [5] made the case for more training of peer reviewers before they are allowed to review in such short time frames to avoid weakening of scientific evidence. El-Menyar et al [4] argued about the necessity to uphold research ethics and best practices in fast-track processes and highlighted the scarcity of original research data, which often led to resources being reused, carrying forward flaws and inaccuracies that shaped public opinion and policies [4,9]. Glasziou [10] pointed out that the COVID-19 research corpus consists largely of preprints and duplicate studies, and Bero et al [11] drew attention to the decreased trustworthiness and validity of less rigorously reviewed COVID-19 research.

A discussion on the real repercussions of an overwhelming focus on COVID-19 research for scientometrics, such as that of the journal impact factor (IF), lacks in the existing literature. Given the role of these measures as benchmarks of research productivity and scholarly impact, editorial practices in favor of fast-track COVID-19 research output have fueled critiques, which view these as attempts to artificially inflate metrics at the potential expense of research quality.

Journal IF constitutes the principal approach to assess scholarly impact in modern research. This appraisal often guides scholars to select where to submit their research and funding bodies to decide where to allocate their resources. Considering the surge of COVID-19 research from the start of the pandemic, a crucial question arises on its influence upon the *gold standard of scholarly assessment* in journals of highest rank. We focused on 6 exemplar high-impact medical journals (Annals of Internal Medicine [Annals], The British Medical Journal [The BMJ], Journal of the American Medical Association [JAMA], The Lancet, Nature Medicine [NatMed], and The New England Journal of Medicine [NEJM]). The aim of our study was to comprehensively compare the productivity trends of COVID-19 and non–COVID-19 literature and track their evolution and scholarly impact across 3 consecutive calendar years.

## Methods

### Data Collection

To fulfil the purpose of our study, we selected 6 high-impact medical journals, namely Annals, The BMJ, JAMA, The Lancet, NatMed, and NEJM. We conducted a comprehensive search of the literature using the Web of Science database for manuscripts published between January 1, 2019, and December 31, 2021. To distinguish between COVID-19 and non–COVID-19 publications, we filtered manuscripts based on their title, abstract, or keywords using the following terms: “COVID-19” OR “SARS-COV2” OR “Coronavirus” OR “2019-nCoV.” Citation counts for each manuscript were retrieved using the Clarivate report function. The search of the literature was performed on a single day to reduce daily updates of the database. Manuscripts were restricted to peer-reviewed original research and review articles. No further exclusion criteria were applied to our search.

### Data Processing

Calculation of a journal’s IF in our study was based on the ratio between the number of citations and manuscripts published in a given journal over a single year for that journal.



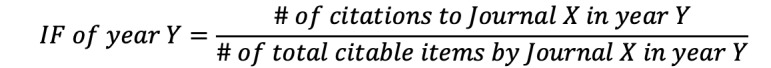



This approach was employed to enhance the temporal resolution of the analysis of scholarly influence from journals in publishing. To assess the effect of COVID-19 and non–COVID-19 literature on scholarly impact of these journals, we initially tracked the evolution of their IFs yearly from 2019 to 2021. We then calculated the percentage change in IFs year on year. Thereafter, we estimated the citation probability of any given COVID-19 and non–COVID-19 publication by journal amid the whole duration. These were expressed as normal distributions and calculated using the normal distribution function (NORMDIST) in Microsoft Excel 2016. On a more granular level, we estimated the publication and citation rate of COVID-19 and non–COVID-19 manuscripts on a monthly basis. Statistical significance was established as *P*<.05, differences in means were examined using a paired sample *t* test (two-tailed), and differences in distribution curves were assessed using an independent *z* test (two-tailed). Statistical analysis was performed using Microsoft Excel 2016 and SPSS statistics software, Version 28.0 (IBM Corp).

## Results

### Journal Impact Factors

**Figure 1:**
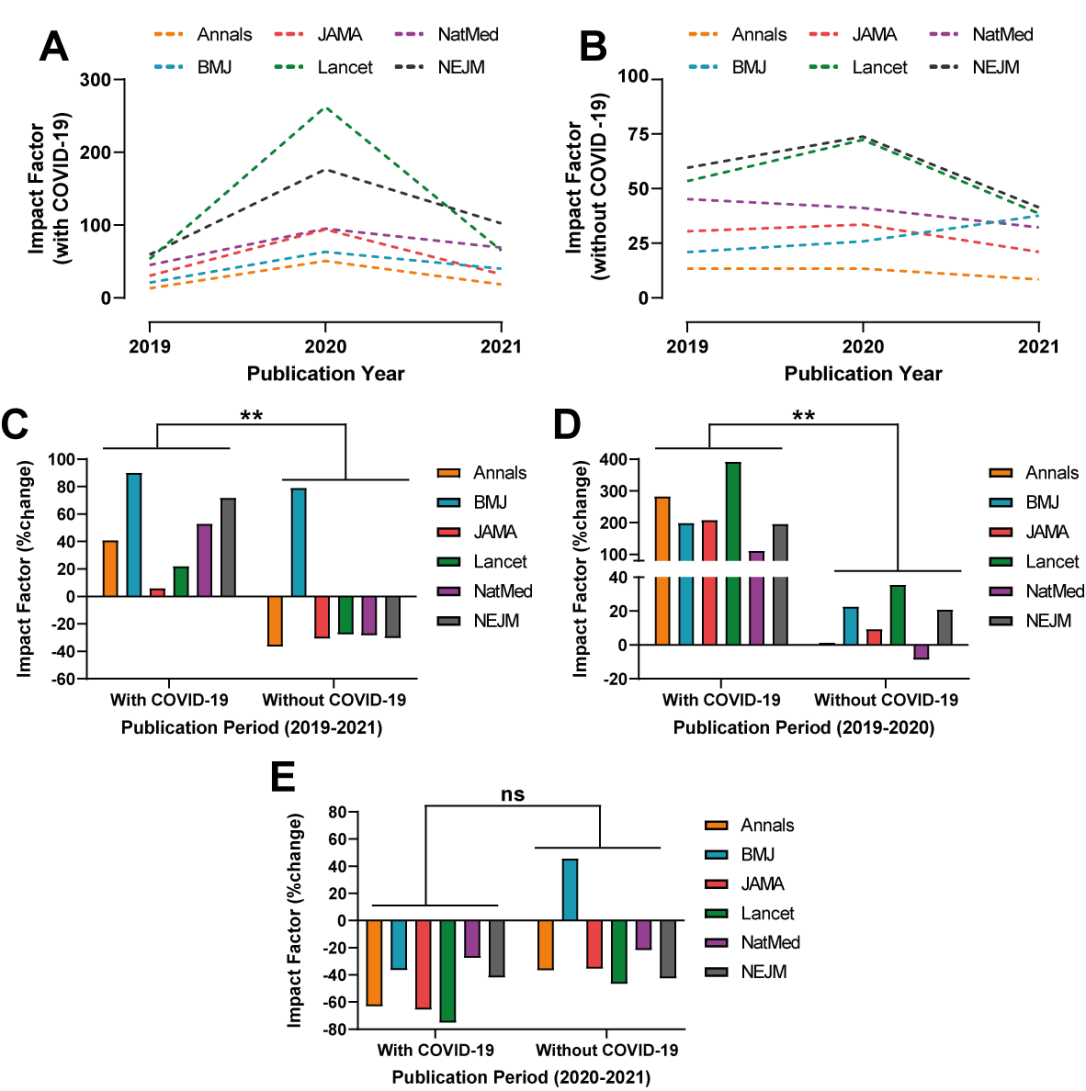
Annual impact factor of 6 high-impact medical journals (Annals of Internal Medicine [Annals], The British Medical Journal [BMJ], Journal of the American Medical Association [JAMA], The Lancet, Nature Medicine [NatMed], and The New England Journal of Medicine [NEJM]) based on (A) manuscripts with and (B) without COVID-19 publications between 2019 and 2021. Changes in annual impact factor comparing manuscripts (C-E) with and without COVID-19 publications between 2019 and 2021. ns: not significant; **P<.01.

The IFs of all 6 high-impact medical journals significantly increased for manuscripts including COVID-19 published from 2019 to 2020 (*P*=.002; Annals: 283%; The BMJ: 199%; JAMA: 208%; The Lancet: 392%; NatMed: 111%; and NEJM: 196%) and to 2021 (*P*=.007; Annals: 41%; The BMJ: 90%; JAMA: 6%, The Lancet: 22%; NatMed: 53%; and NEJM: 72%), when accounting for non–COVID-19 ones (Figure 1 and Table S1-2 in Multimedia Appendix 1). During the former period, The Lancet and Annals experienced the highest increase with a change in IF of 392% and 283% (as opposed to 36% and 1%), respectively. An exception to this trend was NatMed, which saw a decrease in IF of 9% (as opposed to 111%). During the latter period, a more moderate increase was observed across all journals and most prominently of 90% and 72% (as opposed to 79% and -31%) in The BMJ and NEJM, respectively. Notably, The BMJ was the only to experience sustained increase in IF from 2019 to 2020 and 2021. No significant changes were observed from 2020 to 2021 (*P*=.06).

### Probability of Citations

**Figure 2:**
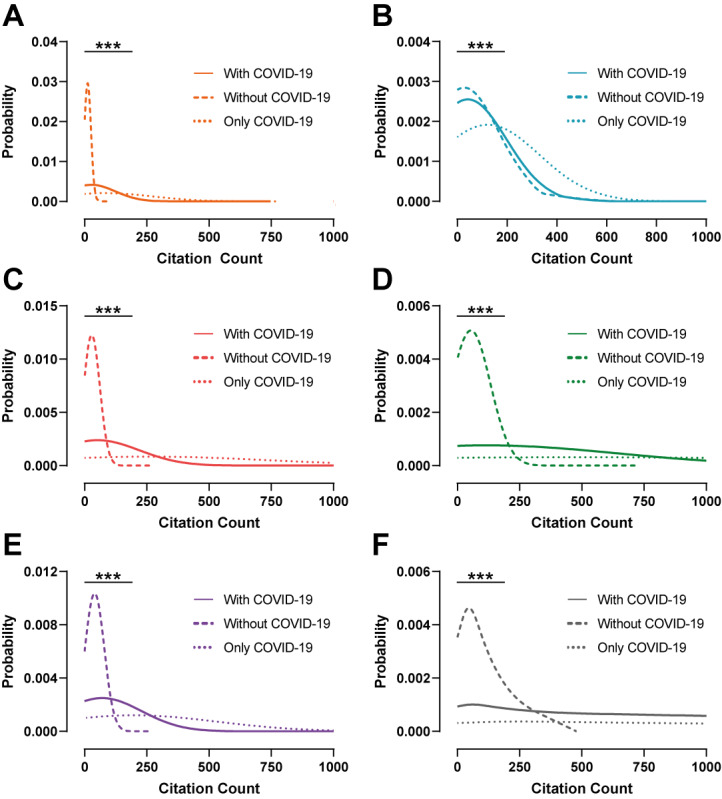
Probability distributions of time-adjusted citation count for 6 high-impact medical journals—(A) Annals of Internal Medicine, (B) The British Medical Journal, (C) Journal of the American Medical Association, (D) The Lancet, (E) Nature Medicine, and (F) The New England Journal of Medicine—based on non–COVID-19, COVID-19–only, and combined publications between 2019 and 2021. ***P<.001.

**Figure 3:**
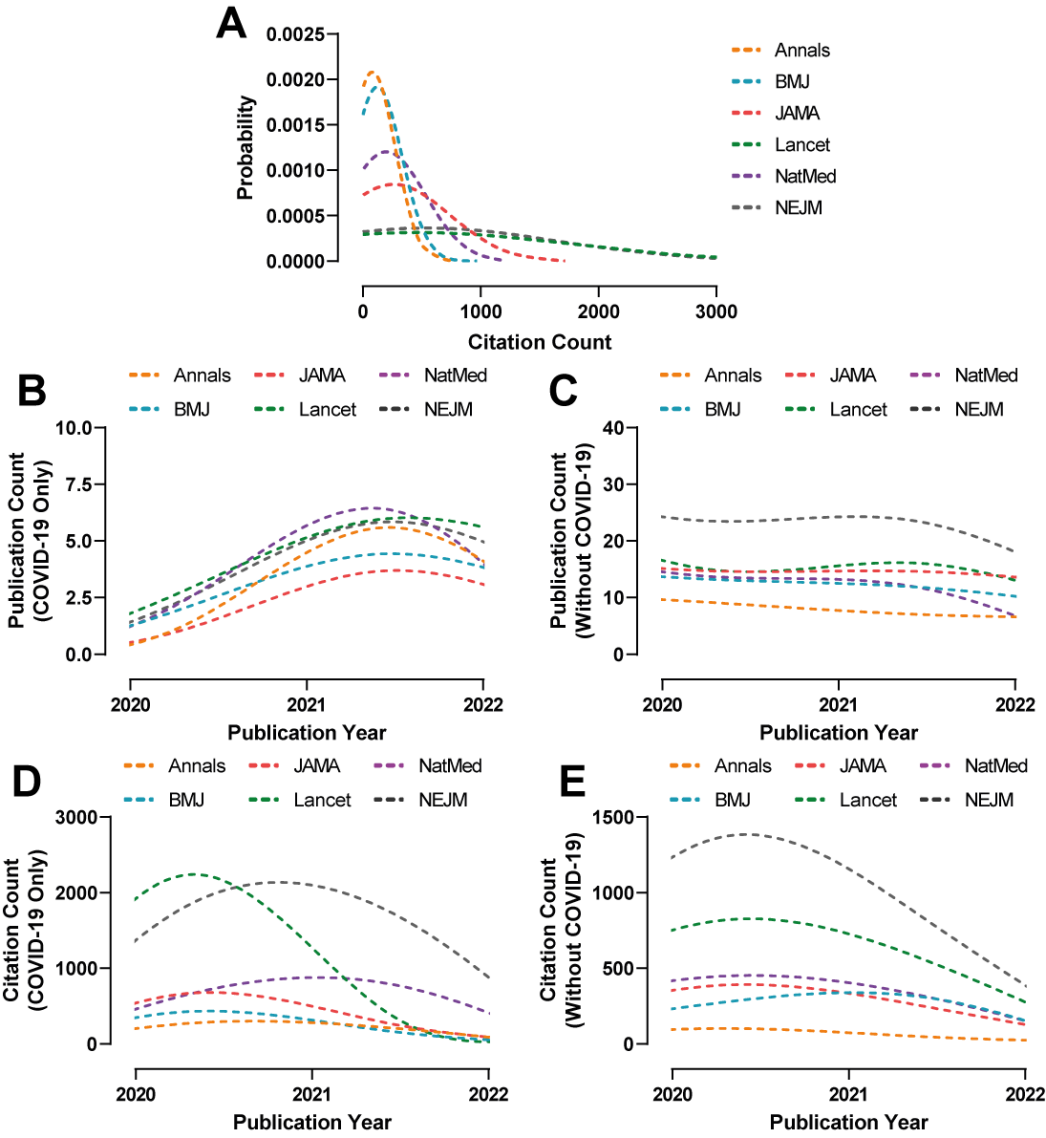
Probability distributions of (A) time-adjusted citation count across 6 high-impact medical journals (Annals of Internal Medicine [Annals], The British Medical Journal [BMJ], Journal of the American Medical Association [JAMA], The Lancet, Nature Medicine [NatMed], and The New England Journal of Medicine [NEJM]) based on COVID-19–only publications between 2019 and 2021. Publication and citation rates based on (B and D) COVID-19–only and (C and E) non–COVID-19 publications between 2020 and 2021.

The probability of highly cited manuscripts published between 2019 and 2021 across all journals was significantly increased for COVID-19 manuscripts compared to non–COVID-19 ones (Annals: *z*=3.4, *P*<.001; The BMJ: *z*=4.0, *P*<.001; JAMA: *z*=3.8, *P*<.001; The Lancet: *z*=3.5, *P*<.001; NatMed: *z*=5.2, *P*<.001; and NEJM: *z*=4.7, *P*<.001; Figure 2 and Table S3 in Multimedia Appendix 1). The highest citation probability was seen in manuscripts published in NatMed (*z*=5.2) during this period. The likelihood of highly cited manuscripts was visually increased across all journals except in that of The BMJ, when considering manuscripts with COVID-19 against those without (Figure 2). Equally, the probability of highly cited COVID-19 manuscripts published during 2019-2021 appeared highest in The Lancet and NEJM compared to the majority of the remaining journals (Figure 3A and Table S4 in Multimedia Appendix 1).

### Rate of Publications and Citations

The publication rate of COVID-19 manuscripts across all journals saw an increase between 2020 and 2021 with a peak by the second quarter of 2021 (Figure 3B). By contrast, the publication rate of non–COVID-19 manuscripts saw a moderate decrease throughout the elapsed duration (Figure 3C). Moreover, the citation rate of COVID-19 manuscripts peaked in the first 2 quarters of 2020 and strongly subsided afterward (Figure 3D). Conversely, non–COVID-19 manuscripts saw a continuous and extensive downward decrease in their citation rate from the start (Figure 3E).

## Discussion

### Principal Findings

Our study showed a significant increase in IF change across 6 high-impact medical journals (Annals, The BMJ, JAMA, The Lancet, NatMed, and NEJM) based on publications including COVID-19 manuscripts from 2019 to 2020 and to 2021, when compared to non–COVID-19 ones. The probability of highly cited manuscripts was significantly increased in COVID-19 manuscripts across most journals and throughout the entire duration, when compared to non–COVID-19 ones. The citation rate for COVID-19 publications peaked by the second quarter of 2020 and that of the publication rate approximately a year later.

### Interpretation of Findings

Our results reflect the capacity of the scientific community to respond against a global health emergency with high-impact publications on COVID-19 at an exponentially expanding rate. With high hopes for a breakthrough, scientists have indeed rushed to publish positive results on the disease [12]. However, this raises concerns whether scientific standards are being met both by researchers and the journals [13]. High-impact medical journals, including The Lancet, Nature, and JAMA embarked on a rapid peer-review initiative to accelerate the dissemination of COVID-19 manuscripts to the public and across the scientific community [14-16]. Nature explicitly invited researchers to shorten review times and decided to reduce the publication of non–COVID-19 content. JAMA expedited the publication of COVID-19 manuscripts within 10 to 12 days from submission [14,15]. A later analysis further confirmed that among other journals, The Lancet and Nature shortened their review processes for COVID-19 articles by almost two-thirds for the duration of the pandemic, when compared to non–COVID-19 submissions [17]. Another report showed that when the quality of peer-reviewed COVID-19 publications was assessed in the 3 most influential medical journals (ie, The Lancet, NEJM, and JAMA), high rates of retraction, withdrawal, or expression of concern were observed [18,19].

Fast-track publications practices have frequently been scrutinized for the rigidity of the research output. This underlies concerns about the quality control of the external peer review and internal editorial evaluation, thorough revision by authors, and journal editing of the manuscript. Most notably, The Lancet and NEJM came under intense fire during the second half of 2020 due to the publishing of false data in a highly influential study regarding the benefit of hydroxychloroquine or chloroquine for the treatment of COVID-19. This information found its way under the public spotlight causing a controversial deluge, leading to its retraction and a barrage of criticism at the integrity and quality of the research and its peer reviewing [20]. Notably, The Lancet has now reflected on the risks of rushed review processes employed as part of their early action against the pandemic and reiterated the need to “slow down” in their publication processes [16]. Nevertheless, a scarcity of explicit information regarding other high-impact medical journals, including The BMJ, NEJM, and Annals, remains. It would be no surprise that similar recorded patterns for COVID-19 publications in these journals could have been attributed to the expedited reviewing processes in an attempt to ease submission bottlenecks.

The growing concern that editorial practices can be as much responsible for the influx in publications as the heightened popularity of the topic among the academic community becomes evident. The attributed responsibility on editorial processes is mainly based on the asymmetrical treatment of COVID-19 research and the consequential encouragement of scientists to focus on COVID-19 by journals. These two acts invite certain types of research by making the route to publication more certain and less time intensive.

There are bearing implications to the potential inflation of journal metrics of research productivity and scholarly impact, such as IF. Journal IFs are commonly used by educational or research groups and various funding bodies to make decisions on the promotion of research proposals, grant applications, but also the awarding of positions to individuals and even salary considerations. In a sense, they provide a way to gauge a scientist or research group’s academic value, or an academic journal’s scientific rigor. Journal IFs, parallel to money, constitute a value system of scholarly influence. To maintain their value across time, they need to rely on stable and transparent processes that remain intact and are always faithfully followed. For academic journals, the main mechanism that controls publication rates and incentivizes research quality is a well-established and thorough peer-reviewing process. Similar to how currency manipulation works, when peer reviewing is altered, there is a risk of distorting the value of and trust in journal IFs as perceived by academia and society.

Apart from the obvious loss of confidence toward the procedures employed by highly reputable publishers, academic journals also face the risk of losing the interest of researchers in publishing to other competitors, and this might be damaging from a business perspective. COVID-19 articles can be a contributing factor to this phenomenon which is exacerbated when fast-track reviewing is made a priority. The lack of transparency and information on how and where exactly fast-track reviewing was implemented during the pandemic magnifies this issue as there is no real way for external parties to assess how much of this is artificially driven. This sows confusion among scholars on how to evaluate the quality of published research and may encourage authors to publish in journals of *predatory* behavior.

Another disservice that journal IF inflation and sudden changes in standards might cause is putting honest and hard-working researchers at a disadvantage. Shortened and sometimes less rigorous peer-review processes, combined with the observed surge in preprints, opinion pieces, and commentaries, while by no means unimportant, increase scientific noise and can waste resources that could be used in a lengthier but more impactful research. The rearrangement of peer reviewing might also benefit authors who are willing to exploit the system in order to inflate their productivity metrics and get an edge over colleagues who are less inclined to take advantage of the *hype*. This can reinforce a deluge of COVID-19 submissions of worrying quality as increasingly more researchers *get the trick* and do not want to miss out on the effortless opportunity to transform their career.

### Limitations

Our study was prone to various inherent limitations. Assessment of IF by year can provide an enhanced temporal resolution of the scholarly influence presented by journals from their research output. However, overtime citations become inflated, and calculating year-specific IFs becomes challenging for a retrospective analysis. To overcome this, we applied a time adjustment on the citations count based on the time elapsed from the start of the search up to date. However, we were not able to account for any traction cycles or short-term events that articles might have experienced over time. Although the IF of all included journals in our study was affected symmetrically by this inherent pitfall, it is likely that the derived yearly IFs were underestimated, especially in articles published at later years. Nevertheless, this phenomenon portrays the crudeness and imperfect abstraction of IF in gaining a more granular investigation. Similarly, COVID-19 manuscripts were restricted to article and review types without taking into consideration related editorials, opinions, or commentaries that constituted a significant portion of the surge in COVID-19 publications from the start. In the same manner, the protocol of data acquisition employed to collect manuscript count was limited to a single database (ie, Web of Science), which could have consequently magnified the quantity of eligible publications. Lastly, a manual screening of the derived publications was not possible, which led to an automatic filtering based on title, abstract, or keywords that best describe COVID-19 terminology. Hence, we could not establish in full whether the retrieved manuscripts indeed focused on COVID-19 and not on other research domains related to it. Taken together, our results and the conclusions derived may be considered more conservative and should be interpreted with caution.

### Conclusions

The rise of COVID-19 has resulted in a surge of scientific production across all areas of knowledge globally. Our findings ultimately demonstrated that the IF, likelihood of being highly cited, and publication and citation rates of manuscripts published across 6 high impact medical journals (Annals, The BMJ, JAMA, The Lancet, NatMed, and NEJM), between 2019 and 2021, were positively skewed by COVID-19 manuscripts. The eruption of COVID-19 publications reinforced the capacity of the scientific community to step up to the challenge, but casted doubt on the reliability of highly susceptible IFs—as shown here—in evaluating scholarly impact. The loss of trust on journal IFs as measures of scientific rigor and confidence in the procedures employed by highly influential publishers may incentivize a culture of exploitation by researchers and journals against the scientific process.

## Appendix

Multimedia Appendix 1 Supplementary tables.

## Abbreviations

Annals: Annals of Internal Medicine
IF: impact factor
JAMA: Journal of the American Medical Association
NatMed: Nature Medicine
NEJM: The New England Journal of Medicine
The BMJ: The British Medical Journal
