# Supplementary material for: How COVID-19 Affected the Journal Impact Factor of High Impact Medical Journals: Bibliometric Analysis
Source: J Med Internet Res. 2022 Dec 21;24(12):e43089. doi: 10.2196/43089 (PMC9778719; doi:10.2196/43089)
Supplement: Multimedia Appendix 1 [file jmir_v24i12e43089_app1.docx]

**Multimedia Appendix 1**

**Table S1.** Impact factor descriptives for six high impact medical journals (Annals of Internal Medicine [Annals], The British Medical Journal [BMJ], Journal of the American Medical Association [JAMA], The Lancet, Nature Medicine [NatMed], and The New England Journal of Medicine [NEJM]) based on manuscripts with and without COVID-19 publications between 2019 and 2021. Values are presented as annual impact factor (publication count; citation count).

| **Journal** | **With COVID-19** | | | **Without COVID-19** | | |
| --- | --- | --- | --- | --- | --- | --- |
|  | **2019** | **2020** | **2021** | **2019** | **2020** | **2021** |
| Annals | 13.2  (130; 1720) | 50.6  (131; 6628) | 18.6  (136; 2529) | 13.2  (130; 1720) | 13.4  (101; 1353) | 8.5  (77; 653) |
| BMJ | 21.1  (179; 3773) | 63.1  (191; 12,052) | 40.1  (179; 7176) | 21.1  (179; 3773) | 25.9  (154; 3980) | 37.6  (132; 4970) |
| JAMA | 30.5  (200; 6096) | 94.0  (196; 18,427) | 32.3  (207; 6689) | 30.5  (200; 6096) | 33.3  (172; 5734) | 21.5  (171; 3678) |
| Lancet | 53.4  (272; 14,516) | 262.4  (212; 55,630) | 65.2  (249; 16,238) | 53.4  (272; 14,516) | 72.4  (164; 11,880) | 38.7  (184; 7115) |
| NatMed | 45.1  (202; 9107) | 95.2  (204; 19,431) | 69.0  (216; 14,906) | 45.1  (202; 9107) | 41.2  (159; 6557) | 32.3  (145; 4678) |
| NEJM | 59.5  (328; 19,522) | 176.4  (331; 58,378) | 102.3  (326; 33,353) | 59.5  (328; 19,522) | 72.0  (281; 20,228) | 41.3  (265; 10,946) |

**Table S2.** Changes in annual impact factor for six high impact medical (Annals of Internal Medicine [Annals], The British Medical Journal [BMJ], Journal of the American Medical Association [JAMA], The Lancet, Nature Medicine [NatMed], and The New England Journal of Medicine [NEJM]) based on manuscripts with and without COVID-19 publications between 2019 and 2021.

| **Journal** | **With COVID-19** | | | **Without COVID-19** | | |
| --- | --- | --- | --- | --- | --- | --- |
|  | **2019 – 2020** | **2020 – 2021** | **2019 – 2021** | **2019 – 2020** | **2020 – 2021** | **2019 – 2021** |
| Annals | 282.5% | -63.2% | 40.9% | 1.3% | -36.7% | -36.6% |
| BMJ | 199.3% | -36.5% | 90.0% | 22.6% | 45.7% | 79.0% |
| JAMA | 208.4% | -65.6% | 5.9% | 9.4% | -35.5% | -30.8% |
| Lancet | 391.7% | -75.1% | 22.1% | 35.7% | -46.6% | -27.7% |
| NatMed | 111.3% | -27.5% | 53.0% | -8.5% | -21.8% | -28.5% |
| NEJM | 196.3% | -42.0% | 71.9% | 20.9% | -42.6% | -30.5% |

**Table S3.** Z-test analysis on the probability distributions of time-adjusted citation count for six high impact medical journals (Annals of Internal Medicine [Annals], The British Medical Journal [BMJ], Journal of the American Medical Association [JAMA], The Lancet, Nature Medicine [NatMed], and The New England Journal of Medicine [NEJM]) comparing COVID-19 and non-COVID-19 publications between 2019 and 2021.

| **Journal** | **COVID-19** | | **Without COVID-19** | | **Z-Test** | |
| --- | --- | --- | --- | --- | --- | --- |
|  | **Citations^a^** | **Manuscripts^b^** | **Citations^a^** | **Manuscripts^b^** | ***Z*** | ***P*** |
| Annals | 80.3 | 89 | 12.1 | 308 | 3.4 | <.001 |
| BMJ | 122.4 | 84 | 27.4 | 465 | 4.0 | <.001 |
| JAMA | 261.7 | 60 | 28.6 | 543 | 3.8 | <.001 |
| Lancet | 467.9 | 113 | 54.0 | 620 | 3.5 | <.001 |
| NatMed | 199.2 | 116 | 40.2 | 506 | 5.2 | <.001 |
| NEJM | 551.2 | 109 | 58.0 | 874 | 4.7 | <.001 |

**^a^**Mean count

**^b^**Observations count.

**Table S4.** Z-test analysis on the probability distributions of time-adjusted citation count of COVID-19 publications comparing six high impact medical journals (Annals of Internal Medicine [Annals], The British Medical Journal [BMJ], Journal of the American Medical Association [JAMA], The Lancet, Nature Medicine [NatMed], and The New England Journal of Medicine [NEJM]) between 2019 and 2021.

| **Journal** | Annals | BMJ | JAMA | Lancet | NatMed | NEJM |
| --- | --- | --- | --- | --- | --- | --- |
| Annals |  | *Z*=-1.4  *P*=.2 | *Z*=-2.8  *P*=.005 | *Z*=-3.2  *P*=.001 | *Z*=-3.2  *P*=.001 | *Z*=-4.4  *P<*.001 |
| BMJ | *Z*=1.4  *P*=.2 |  | *Z*=-2.1  *P*=.03 | *Z*=-2.8  *P*=.004 | *Z*=-2.0  *P*=.04 | *Z*=-4.0  *P<*.001 |
| JAMA | *Z*=2.8  *P*=.005 | *Z*=2.1  *P*=.03 |  | *Z*=-1.5  *P*=.1 | *Z*=0.9  *P*=.4 | *Z*=-2.4  *P*=.02 |
| Lancet | *Z*=3.2  *P*=.001 | *Z*=2.8  *P*=.004 | *Z*=1.5  *P*=.1 |  | *Z*=2.2  *P*=.03 | *Z*=-0.5  *P*=.6 |
| NatMed | *Z*=3.2  *P*=.001 | *Z*=2.0  *P*=.04 | *Z*=-0.9  *P*=.4 | *Z*=-2.2  *P*=.03 |  | *Z*=-3.2  *P*=.001 |
| NEJM | *Z*=4.4  *P*<.001 | *Z*=4.0  *P*<.001 | *Z*=2.4  *P*=.02 | *Z*=0.5  *P*=.6 | *Z*=3.2  *P*=.001 |  |
